# Novel D-A-D Fluorescent Dyes Based on 9-(*p*-Tolyl)-2,3,4,4a,9,9a-hexahydro-1*H*-carbazole as a Donor Unit for Solution-Processed Organic Light-Emitting-Diodes

**DOI:** 10.3390/molecules26102872

**Published:** 2021-05-12

**Authors:** Vladislav M. Korshunov, Maxim S. Mikhailov, Timofey N. Chmovzh, Andrey A. Vashchenko, Nikita S. Gudim, Lyudmila V. Mikhalchenko, Ilya V. Taydakov, Oleg A. Rakitin

**Affiliations:** 1 P. N. Lebedev Physical Institute of the Russian Academy of Sciences, 53 Leninskiy Prospect, 119991 Moscow, Russia; andrewx@mail.ru (A.A.V.); taidakov@mail.ru (I.V.T.); 2 Faculty of Fundamental Sciences, Bauman Moscow State Technical University, 105005 Moscow, Russia; 3 N. D. Zelinsky Institute of Organic Chemistry, Russian Academy of Sciences, Leninsky Prospect, 119991 Moscow, Russia; mihailov_maxim_s@mail.ru (M.S.M.); tim1661@yandex.ru (T.N.C.); nikitosgudim@gmail.com (N.S.G.); mlv@ioc.ac.ru (L.V.M.); 4 Nanotechnology Education and Research Center, South Ural State University, 454080 Chelyabinsk, Russia; 5 Academic Department of Innovational Materials and Technologies Chemistry, Plekhanov Russian University of Economics, 117997 Moscow, Russia

**Keywords:** 2,1,3-benzochalcogenadiazoles, 2,3,4,4a,9,9a-hexahydro-1*H*-carbazole, D-A-D dyes, luminescence, OLEDs

## Abstract

New fluorescent D-A-D dyes containing 9-(*p*-tolyl)-2,3,4,4a,9,9a-hexahydro-1*H*-carbazole as a donor unit and 2,1,3-benzochalcogenadiazoles as an electron-withdrawing group were synthesized. The photoluminescent and electroluminescent properties of novel dyes for fluorescent OLED application were investigated. It was demonstrated that the replacement of lightweight heteroatoms by heavier ones enables the fine tuning of the maximum emission without significantly reducing the luminescence quantum yield. The maximum quantum yield value of 62.6% for derivatives based on 2,1,3-benzoxadiazole (**1a**) in cyclohexane was achieved. Two devices with the architecture of glass/ITO/PEDOT-PSS/poly-TPD/EML/TPBi/LiF/Al (EML = emitting layer) were fabricated to check the suitability of the synthesized compounds as a single active emission layer in OLED. These OLEDs exhibited clear red electroluminescence of the dyes with the maximum current efficiency of 0.85 Cd/A.

## 1. Introduction

In recent decades, there has been an active search and study of new donor-acceptor (D-A) organic compounds. This interest is due to a wide range of practical problems associated with the development of electronic devices, such as organic light-emitting diodes (OLED) [1,2,3,4], dye-synthesized solar cells which convert solar energy into electrical energy (DSSC) [5,6], and electronic switches [7,8]. Among the most significant applications of compounds from this class is the possibility of their use as an emitting material for Li-Fi technologies [9]. Particular attention is paid to structures of the D-A-D type, since the introduction of an additional donor improves the properties of the intramolecular charge transfer (ICT) mechanism [10,11]. Interest in dyes from this class is primarily associated with the ability to vary donor and acceptor molecular fragments. Thus, it becomes possible to fine tune the energies of the highest occupied molecular orbital (HOMO) and the lowest unoccupied molecular orbital (LUMO), and hence the positions of the absorption and luminescence maxima. As a result, it is possible to purposefully synthesize materials capable of luminescence in the entire visible range of the spectrum, as well as in the near infrared part of the spectrum.

It is known that compounds based on the acceptor fragment of 2,1,3-benzochalcogenadiazole exhibit bright fluorescence in the orange-red [12] and near-infrared region of the spectrum [13]. In addition, compounds with such a fragment are actively used to create active light-emitting layers in OLED [14].

It is known from the literature that carbazole derivatives are among the most widely used donor fragments [15]. Such substances exhibit not only high luminescent characteristics, but also have increased thermal stability. However, often, due to the formation of π-π stacking between aromatic rings, the undesirable aggregation of molecules occurs, causing quenching of luminescence [16]. Among the ways to prevent unwanted aggregation is to switch to donor fragments with bulky aliphatic groups attached [17]. We recently synthesized a new donor building block based on indoline moiety—9-(*p*-tolyl)-2,3,4,4a,9,9a-hexahydro-1*H*-carbazole [18]—which has a high electron density and contains a flexible aliphatic frame. Thus, building blocks of this class have been successfully used to obtain highly efficient dyes for organic solar cells [18,19,20]. However, to date, there have been no data in the literature on the study of the luminescence characteristics of compounds based on indoline derivatives.

**Figure 1:**
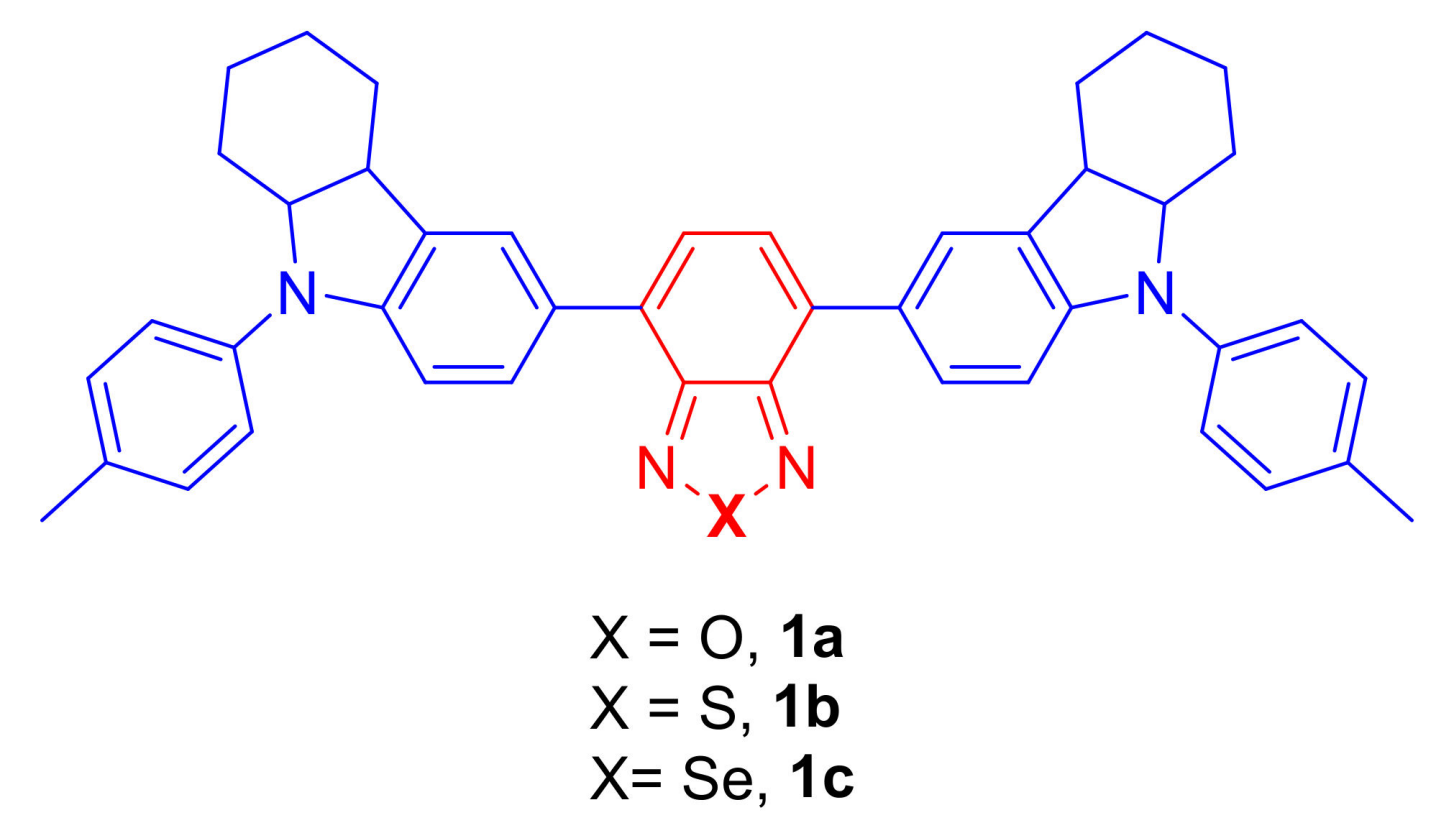
Chemical structures of the dyes synthesized.

In the present work, new compounds of the DAD type based on 9-(*p*-tolyl)-2,3,4,4a,9,9a-hexahydro-1*H*-carbazole, as an electron donor and 1,3-benzochalcogenadiazoles with various chalcogen atoms (oxygen, sulfur, and selenium) as an acceptor were synthesized and investigated (Figure 1). Different heteroatoms were used in order to establish the influence of their nature on the photo- and electroluminescent characteristics of the dyes. The 9-(*p*-tolyl)-2,3,4,4a,9,9a-hexahydro-1*H*-carbazole moiety as donor was used, since the presence of bulky groups in the donor fragment prevents the formation of undesired π-π stacking.

## 2. Results and Discussion

### 2.1. Synthesis and Characterization

**Scheme 1:**
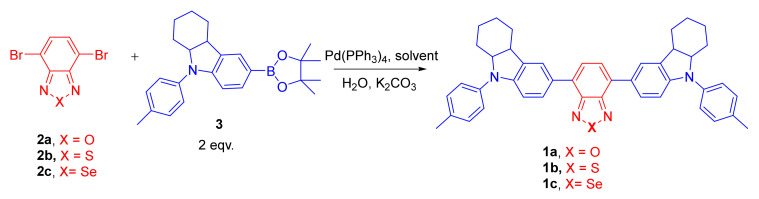
Suzuki–Miyaura cross-coupling reactions of 4,7-dibromobenzo[*c*] [1,2,5]chalcogenadiazoles.

For the synthesis of target molecules of the D-A-D type, we studied the Suzuki–Miyaura cross-coupling reactions of 4,7-dibromobenzo[*c*] [1,2,5]chalcogenadiazoles **2**(**a**–**c**) with two equivalents of the donor boronic ester **3** in the presence of Pd(PPh_3_)_4_ as a catalyst and aqueous solution K_2_CO_3_ (Scheme 1). It was shown that when the reaction was carried out in THF, the formation of mainly monosubstituted products occurred in moderate yields, while the yields of bis-derivatives **1**(**a**–**c**) did not exceed 25% (Table 1, entries 1–3). In contrast, replacing THF with a higher boiling dioxane within 24 h resulted in the replacement of both bromine atoms with donor fragments to obtain disubstituted products in high yields (80 to 85%) (Table 1, entries 3–6). All dyes were purified by column chromatography before measurement of the physical and electrochemical properties.

### 2.2. Photophysical and Electrochemical Properties

To determine the energy of the boundary orbitals of compounds **1**(**a**–**c**) (Table 2), we used the values of the formal potentials of their oxidation and reduction obtained by the method of cyclic voltammetry (CV). Cyclic voltammograms (see Supplementary Materials Figure S1) were recorded in solutions of anhydrous DMF in an argon atmosphere.

The first stages of electroreduction and electro-oxidation of the compounds **1**(**a**–**c**) were studied at a low potential sweep rate (v = 100 mVs^−1^). The formal oxidation and reduction potentials were determined as the average value of the potentials of the forward and reverse scan peaks for the corresponding redox system: E_ox_ = (E_a_ + E_a_)/2 and E_red_ = (E_a_ + E_a_)/2. For the transition to the absolute scale of potentials, the values of E_ox_ Fc/Fc^+^ and E_red_ Fc/Fc^+^ were calculated relative to the reversible oxidation potential of ferrocene (Fc/Fc^+^) (see Table 2). The energies of the highest occupied molecular orbital (E_HOMO_) and the lowest vacant (E_LUMO_) were calculated by Equations (1) and (2), taking into account [21,22] the value of the absolute oxidation potential of ferrocene −5.1V:

E_HOMO_ (eV) = −|e|(E^ox^ _Fc/Fc+_ + 5.1) (1)

E_LUMO_ (eV) = −|e|(E^red^ _Fc/Fc+_ + 5.1) (2)

It was demonstrated that the replacement of lightweight heteroatoms by heavier ones in the compounds **1**(**a**–**c**) led to a change in HOMO and LUMO energies without clear dependence of each energy on the heteroatom type. Consequently, the energy gap E_g_ is equal to 2.05, 2.14, and 1.81 eV for **1a**, **1b**, and **1c**, respectively.

### 2.3. Photoluminescence Studies

**Figure 2:**
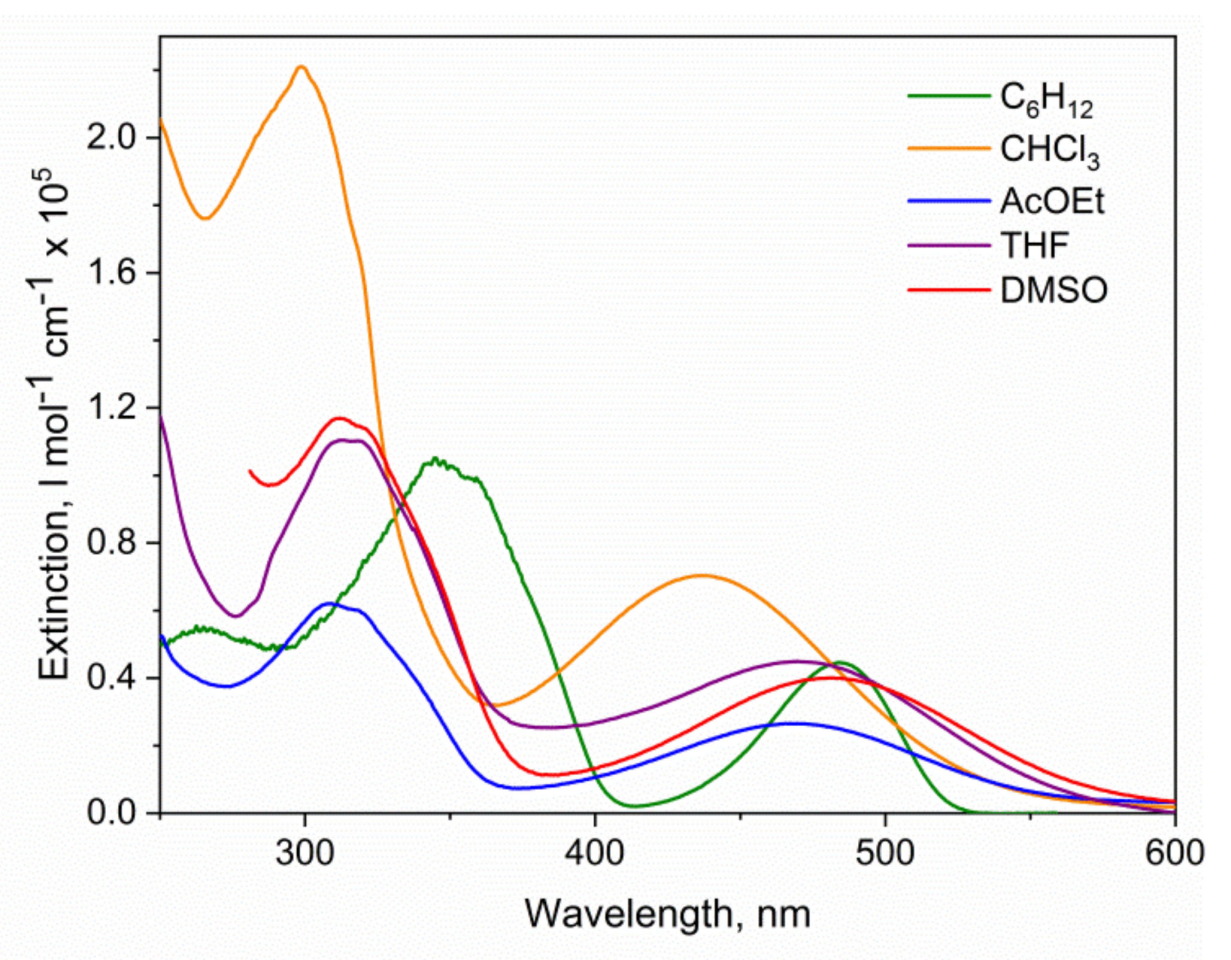
UV–Vis spectra for solutions of compound **1b**.

UV–Vis spectra recorded for the investigated solutions of dye **1b** are presented in Figure 2 and in Supplementary Materials Figure S2. These spectra reveal several absorption bands located at 450–500 nm and 300–350 nm corresponding to the ICT state and π-π* transition, respectively. The investigated compounds demonstrated a noticeable blue shift of maxima in the absorption spectra under increasing solvent polarity. The dependence of the shift of the spectral maximum on the solvent polarity has a more complex shape. Namely, there is an unexpectedly big blue-shift by 50 nm for chloroform (CHCl_3_) solution (λ_abs_ = 485 nm for cyclohexane C_6_H_12_ and λ_abs_ = 437 nm for CHCl_3_).

**Figure 3:**
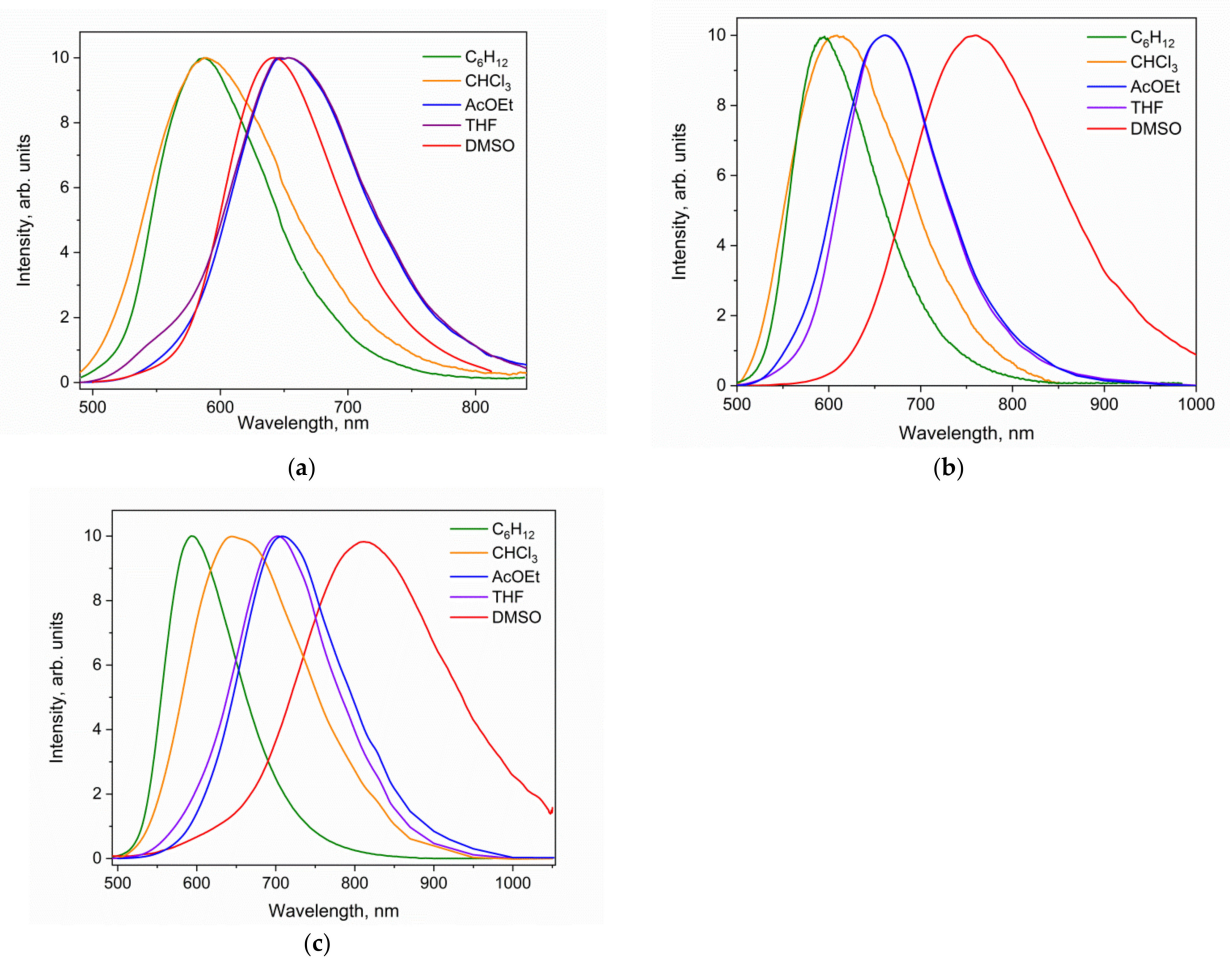
Photoluminescence spectra for investigated compounds **1a** (**a**), **1b** (**b**), and **1c** (**c**) recorded in solutions.

Photoluminescence spectra (PL) for investigated compounds **1a**, **1b**, and **1c** were recorded in various solutions (Figure 3). The compounds exhibited bright luminescence under optical excitation at 360 nm. Two broad spectral bands were observed for solutions of **1a** in chloroform and for **1c** in cyclohexane and chloroform, whereas the last sample, **1b**, demonstrated a single emission band (see Figure 3). The PL spectra have a big full width at half maximum (FWHM) value, around 50–100 nm, with a trend of increasing solvent polarizability (Δf). The bathochromic shift of emission maximum (λ_em_) occurred due to the solvatochromic effect, implying that luminescence has a nature of radiative relaxation of the ICT state of compounds. We observed the fine tuning of emission color in the orange to near-infra red (NIR) spectral range. The maximum wavelength of luminescence shifted from 585 to 660 nm for **1a**, 594 to 761 nm for **1b**, and from 580 to 806 nm for **1c**. As it was demonstrated, the early [23,24] replacement of heteroatom O on Se led to a reduction in the HOMO-LUMO energy gap from 2.05 to 1.81 eV and, therefore, absorption and emission maximum redshifts took place. On the contrary, λ_em_ was still the same for **1b** compounds dissolved in the nonpolar cyclohexane (see Table 3). As can be seen from Figure 3, emission maximum redshifted from cyclohexane to DMSO solution by 220 nm for **1a**, 170 nm for **1b,** and only 54 nm for selenadiazole containing dye **1c**. Hence, we claim that the influence of solvent polarity on the energy of the excited ICT state becomes less pronounced than when transitioning from a light to heavy heteroatom.

**Figure 4:**
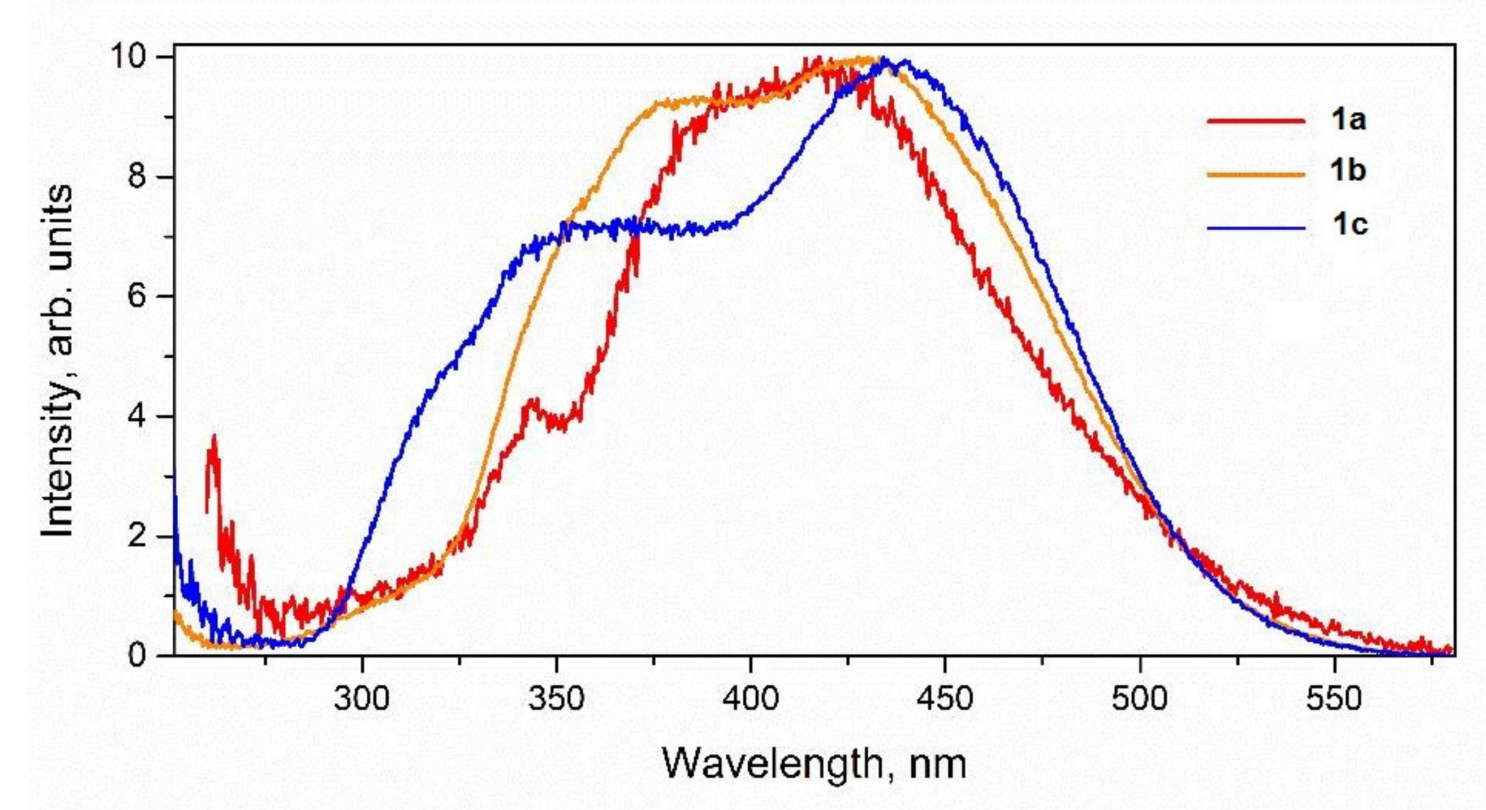
PL excitation spectra recorded with registration at the maxima of emission spectra.

Excitation spectra obtained for chloroform solutions of compounds **1**(**a**–**c**) qualitatively resemble the corresponding optical absorption spectra in the visible and UV regions of the spectrum (see Figure 4). Therefore, we conclude that the luminescence of **1**(**a**–**c**) more efficiently excites via the ICT state than if it excited via π-π* transitions (250–350 nm). To estimate the luminescence efficiency, we recorded PL decays and PL quantum yields (Φ) with an optical excitation at 460 nm. The photoluminescence decays recorded for solutions of compound **1b** are presented in Supplementary Materials Figure S4. The decays were well fitted by the monoexponential function. As seen from Table 3, the observed luminescence lifetimes of **1b** reached the maximum 6.8–6.9 ns for medium polarity solvents, such as ethyl acetate or THF. The **1a** and **1c** dyes demonstrated lifetimes lower than 6 ns with an analogous trend of polarity. However, Φ monotonically reduced from 62.6 to 9% for 1a, from 56 to 11% for 1b, and from 24.7 to 4% for **1c**, while solvent polarity increased. According to our calculations, the reduction of quantum yield occurs rather due to decrease of the probability of the radiative processes (k_r_). Since the error of QY estimation did not exceed 15% of the measured value, it did not have a significant influence on the observed trends.

### 2.4. Theoretical Calculations

**Figure 5:**
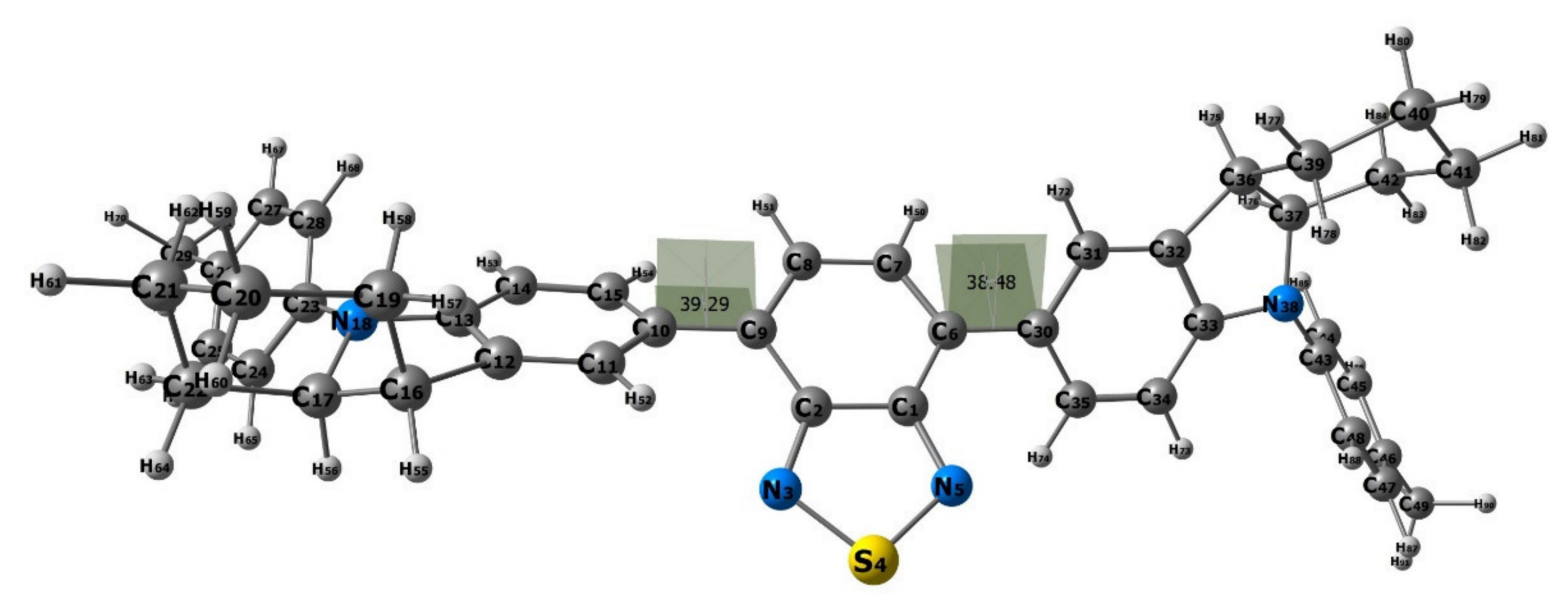
The selected dihedral angles of compound **1b** in THF solvent at ground state.

**Figure 6:**
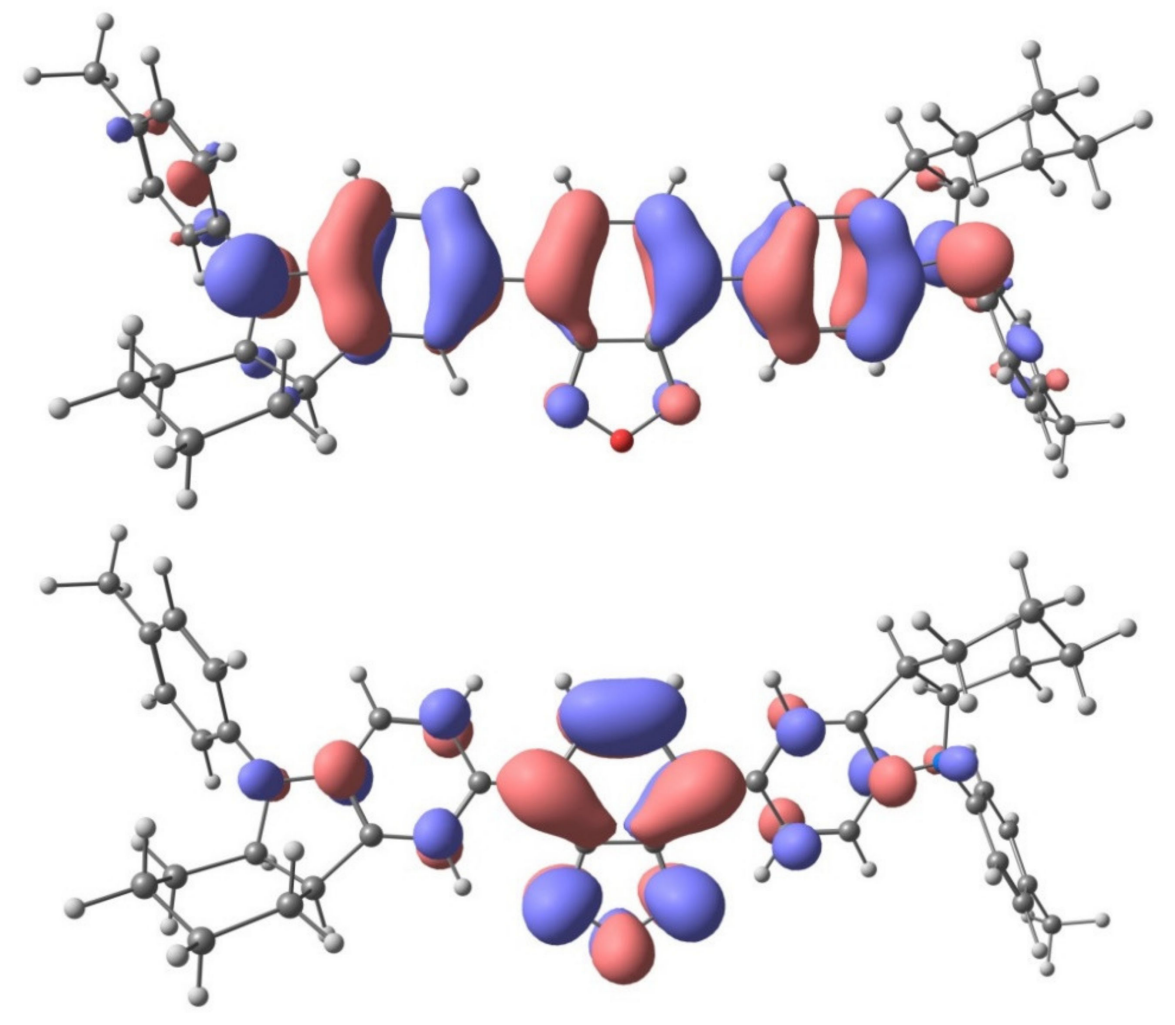
The calculated HOMO (**top**) and LUMO (**bottom**) orbitals for **1a** compound on the geometry of S_1_ state.

DFT and TD-DFT calculations were performed to gain further insight into the electronic properties of the molecules. We observed a rotation around the bond connecting the donor and central acceptor unit (see Figure 5) when we moved from the ground to first excited singlet state. The dihedral angle D(C8,C9,C10,C15) changed from 39.60° by 23.40°, from 39.29° by 18.4°, and from 35.29° by 15.90° for **1a**, **1b**, and **1c**, respectively. The dihedral angle D(C7,C6,C30,C31) had a slightly lower value but demonstrated the same trend. Furthermore, D(C8,C9,C10,C15) had a maximum value 39.6° for **1c**, suggesting the highest reaction barrier between S_1_(LE) and S_1_(ICT) states of the **1c** dye between all molecules under investigation. Consequently, a reduction in the ICT state emission was observed. As a result, biexponential decays of luminescence originating from both LE and ICT states were registered for solutions of this dye. The contribution of the HOMO-LUMO transition with a maximum (93%) in S_0_–S_1_ was the most substantial for all the dyes. The calculated frontier molecular orbitals HOMO and LUMO are depicted in Figure 6. The HOMO was mainly located equally on three benzene fragments of donors and acceptor moieties. Conversely, LUMO was mainly located on the acceptor moiety, suggesting the charge transfer character of HOMO-LUMO transition. From the comparison of LUMO for all dyes, we hypothesize that the replacement of lightweight heteroatoms by heavier ones (O, S, Se) leads to an increase in dipole moment μ_calc_ due to the improvement of acceptor properties of the central acceptor moiety. On the contrary, the oscillator strength value (*f*) decreased from 1.07 **1a** to 0.67 **1c**. The calculated *f* values are in very good agreement with the experimental ones.

The optimized first excited state geometries were used to compute vertical excitation wavelengths. Thus, the calculated absorption maxima correlate well with the first excited singlet state (S_1_) energies experimentally determined as 0-0 phonon transition (see Supplementary Materials Figure S3). Due to the significant geometry distortion caused by rotation around the bonds, connecting each donor and the acceptor moieties in S_1_ state, the calculated vertical excitation wavelengths differ by 90–105 nm in comparison with the maxima (λ_abs_) in optical absorption spectra wavelengths, but had the same trend. Frontier molecular orbitals with the most contribution to the S_0_–S_1_ transition are listed in Table 4. For **1a**, we also calculated the Franck–Condon absorption spectrum. The absorption band in the theoretical Franck–Condon spectrum centered at λ_abs_ = 475 nm closely matched the experimental one at 480 nm. Calculated simulated emission maxima were different to those obtained from the experiments, but with the same trends regarding the replacement of lightweight heteroatoms by heavier ones. Namely, we theoretically and experimentally observed a red shift of the maximum emission for **1**(**a**–**c**).

### 2.5. Electroluminescence

OLED devices were fabricated and examined to test the suitability of novel dyes as emissive layers for OLED. Dyes **1a** and **1b** were selected as emissive materials since their luminescence quantum yields are remarkably higher than that of **1c**. The structures of the fabricated devices were as follows:

ITO/PEDOT-PSS (50 nm)/poly-TPD (20 nm)/**1a** (10 nm)/TPBi (20 nm)/LiF (1 nm)/Al (100 nm)—Structure 1;
ITO/PEDOT-PSS (50 nm)/poly-TPD (20 nm)/**1b** (10 nm)/TPBi (20 nm)/LiF (1 nm)/Al (100 nm)—Structure 2.

**Figure 7:**
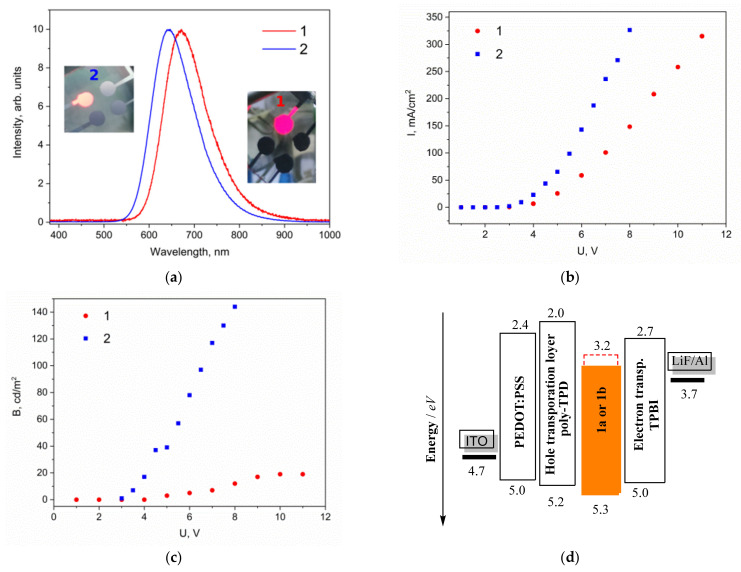
Electroluminescence spectra at 12 V (**a**), density-voltage (**b**), and luminance-voltage (**c**) plots for structures 1 and 2. The schematic diagram of the structures of OLEDs and the energy levels of the materials (**d**).

The electroluminescence spectra were qualitatively similar to the photoluminescence ones. The maximum electroluminescence wavelength (660 and 640 nm) was slightly blueshifted in comparison to photoluminescent λ_em_ by 30 nm, due to the influence of electric fields in OLED devices. They had the emission maxima at 670 and 645 nm for structures 1 and 2, respectively (see Figure 7a). Notably, there was no TPBi fluorescence observed in the UV-blue region of the spectrum. This can be explained by the efficient exciton energy transfer from conductive layers to emissive material. The CIE coordinates of devices 1 and 2 were located at (0.76, 0.32) and (0.65, 0.35). We estimated a relatively low turn-on voltage, i.e., the voltage when the visible electroluminescence appears, of 2.4 V for structure 2 and 3.0 V for structure 1. The highest obtained luminance was 144 Cd/m^2^ for structure 2 at 8 V, whereas the luminance recorded for structure 1 did not exceed 20 Cd/m^2^ (see Figure 7c). However, the relatively high current densities limited the current efficiency and external quantum efficiency values to 0.85 Cd/A and 0.70% at 4.5 V operating voltage for structure 2. Growth of bias voltage provides a decrease of current efficiency and EQE up to 0.45 Cd/A and 0.35% with consequent saturation. In fact, the efficiency of 0.85 Cd/A was comparable with the typical results for solution-processed OLEDs based on fluorescent small molecules [25,26]. The schematic diagram of the structures of OLEDs and the energy levels of the materials is shown in Figure 7d. In [27], the spectral properties of a series of similar compounds with a carbazole donor were investigated. All compounds based on thiadiazole and selenadiazole were characterized by luminescence in the range of 500–600 nm. A molecule similar to our compound with the same acceptor block and a carbazole fragment in the donor part of the molecule was obtained and OLED based on this compound was fabricated and tested [28]. The compound showed orange emissions both in solutions and upon electroexcitation in OLED. Thus, we showed that the use of 9-(*p*-tolyl)-2,3,4,4a,9,9a-hexahydro-1*H*-carbazole instead of a carbazole analog leads to a significant shift in the EL maximum.

## 3. Experimental Details

### 3.1. Materials and Reagents

Chemicals were purchased from commercial sources (Sigma-Aldrich, St. Louis, MO, USA) and used as received. 4,7-Dibromo-2,1,3-benzoxadiazole (**2a**) [29], 4,7-dibromo-2,1,3-benzothiadiazole (**2b**) [30], 4,7-dibromo-2,1,3-benzoselenadiazole (**2c**) [31], and 4,6-(4,4,5,5-tetramethyl-1,3,2-dioxaborolan-2-yl)-9-(*p*-tolyl)-1,2,3,4,4a,9a-hexahydrocarbazole **3** [18] were prepared according to the previously described protocols. All synthetic operations were performed under a dry argon atmosphere. Solvents were purified by distillation over the appropriate drying agents.

### 3.2. Analytical Instruments

^1^H- and ^13^C-NMR spectra were obtained with a Bruker AM-300 NMR spectrometer (Bruker Ltd., Moscow, Russia) (at frequencies of 300.1 and 75.5 MHz, respectively) in CDCl_3_ solutions, with TMS as the standard. *J* values are given in Hz. Multiplicities are assigned as s (singlet), d (doublet), t (triplet), and m (multiplet). MS spectra (EI, 70 eV) were obtained with a Finnigan MAT INCOS 50 instrument (Thermo Finnigan LLC, San Jose, CA, USA). High-resolution mass spectra were measured on a Bruker micrOTOF II instrument using electrospray ionization (ESI). IR spectra were measured with a Bruker “Alpha-T” instrument (Bruker, Billerica, MA, USA) in KBr pellets.

Optical absorption spectra of the investigated dyes were measured at ambient temperature using a JascoV-770 spectrophotometer operating within the 190–2500 nm spectral region. Excitation and emission spectra, as well as the luminescence quantum yield (QY), were measured at ambient temperature in the crystalline phase. For this purpose, a Horiba Jobin Yvon Fluorolog FL3-22 spectrofluorimeter (Kyoto, Japan) equipped with a 450 W xenon lamp emitting within the 250–900 nm spectral range was employed. The luminescence of the samples was detected with a Hamamatsu R928 photomultiplier (Hamamatsu, Japan) operating within 200–850 nm. For the QY measurements, a G8 Spectralon^®^-covered integrating sphere (GMP SA, Renens, Switzerland) was mounted inside the spectrofluorimeter. The sample in a quartz vial was placed into the sphere. All QY measurements were repeated at least three times to achieve the experimental error ± 15%.

### 3.3. Computational Details

Quantum-chemical calculations were performed using the Gaussian 16 Rev A.03 program [32]. DFT (or TD-DFT for excited states) ωB97XD/6-31G(d) level of theory was used for all calculations. In the development of the theoretical models, geometrical parameters from various experimental X-ray structures taken from The Cambridge Structural Database (CCDC) were used as starting points. All calculations were performed in THF (PCM model). The corrected linear-response approach was used for excited states. Cartesian coordinates are given in angstroms; absolute energies for all substances are given in hartrees (see ESI for details). The first excited state of each molecule was optimized and the UV–Vis spectrum was calculated using the Franck–Condon–Herzberg–Teller method. Analysis of vibrational frequencies was performed for all optimized structures. All compounds (both ground and first excited states) were only characterized by real vibrational frequencies. Wave function stability, using STABLE keyword, was also checked for the ground state of each molecule. Absorption wavelength maxima were calculated from the simulated (Franck–Condon–Herzberg–Teller method) UV–Vis absorbance spectrum.

### 3.4. Device Fabrication and Characterization

Poly(3,4-ethylenedioxythiophene):poly(styrenesulfonic acid) (PEDOT:PSS) hole injection material, 2,2′-dimethyl-*N*,*N*′-di-[(1-naphthyl)-*N*,*N*′-diphenyl]-1,1′-biphenyl-4,4′-diamine (α-NPD) hole transport material, 4,4′-bis(*N*-carbazolyl)-1,1′-biphenyl (CBP) host layer material, 1,3,5-tris[*N*-(phenyl)benzimidazole]-benzene (TPBi) electron transport material as well as LiF and Al cathode materials were purchased from Luminescence Technology Corp. (New Taipei City, Taiwan) and used without further purification.

ITO-coated glass substrates were washed in an alcohol solution of KOH followed by ultrasonic treatment in double distilled water for 20 min. The substrates were dried in a flow of dust-free nitrogen. Then, a 50 nm-thick PEDOT:PSS layer was deposited by spin coating, with further annealing in air at 120 °C for 20 min. The substrates were then transferred to an argon-filled glovebox equipped with a Leybold-Heraeus Univex 300 vacuum deposition chamber (Cologne, Germany). OLED active layers were deposited by spin coating of the corresponding dyes. The layers of α-NPD, TPBi, LiF, and Al were deposited by thermal evaporation in a vacuum (residual pressure below 10^−3^ Pa). The deposition thicknesses were controlled by a Leybold Inficon IC-6000 deposition controller (Cologne, Germany). The controller was calibrated by deposition of reference films, with the subsequent measurement of their thicknesses taken with an NT-MDT Integra atomic force microscope (Zelenograd, Russia).

The luminance of the OLED samples was measured by a TKA-PKM luminance meter produced by TKA Scientific Instruments (St. Petersburg, Russia). Current-voltage characteristics were obtained using an automated setup involving a Keitley 6485, Agilent 34401A voltmeter (Solon, OH, USA), and a Motech 2019 power supply (Moscow, Russia). OLED optical power was determined using a Coherent FieldMaxII Laser Power Meter (Santa Clara, CA, USA) with a calibrated photodiode head. Electroluminescence (EL) spectra were obtained with an Ocean Optics Maya 2000 Pro CCD spectrometer (Orlando, FL, USA).

### 3.5. Electrochemical Characterization

Electrochemical measurements were carried out in a dry argon atmosphere using an IPC Pro MF potentiostat (Houten, The Netherlands). The redox properties of compounds were determined using cyclic voltammetry in a three-electrode electrochemical system. A three-electrode system consisting of platinum as the working electrode with an area of 0.8 mm^2^, platinum wire as the counter electrode, and a saturated calomel electrode (SCE) as the reference electrode was employed. The reduction and oxidation potentials were determined in DMF, using 0.1 mol L^−1^ *n*-Bu_4_NClO_4_ as the supporting electrolyte. Cyclic voltammetry (CV) measurements used scan rates of 0.1 V s^−1^. The first reduction/oxidation potentials were referenced to the internal standard redox couple Fc/Fc^+^. Ferrocene was added to each sample solution at the end of the experiment, and employed for calibration.

### 3.6. General Procedure for the Preparation of Bis-Substituted Products ***1**(**a**–**c**)* under Suzuki Coupling Conditions

In a 50 mL round-bottom flask, **3** (281 mg, 0.75 mmol) and 4,7-dibromobenzo-1,2,5-chalcogenadiazole **2** (0.3 mmol) were dissolved in 15 mL of solvent (THF or dioxane), and 2 M K_2_CO_3_ (10 mL) was added. The mixture was degassed for 20 min with a stream of argon, then Pd(PPh_3_)_4_ (42 mg, 5 mmol %) was added. After refluxing for 24 h, the mixture was extracted with CH_2_Cl_2_; organic solvent was removed under the reduced pressure. The residue was purified by column chromatography.

*4,7-Bis(9-(p-tolyl)-2,3,4,4a,9,9a-hexahydro-1H-carbazol-6-yl)benzo[c][1,2,5]oxadiazole* (**1a**)

Dark red solid, yield 164 mg (85%), m.p. 155–157 °C. R_f_ = 0.5 (hexane:ethylacetate, 10:1 (*v*/*v*)). IR, v_max_, cm^−1^: 2927, 2853, 1605, 1513, 1481, 1380, 1268, 1108, 806. ^1^H-NMR (300 MHz, CDCl_3_): 1.49–1.36 (m, 4H), 1.62–1.53 (m, 4H), 1.88–1.72 (m, 6H), 2.00–1.9 (m, 2H), 2.40 (s, 6H), 3.35 (m, 2H), 4.17 (m, 2H), 6.86 (d, *J* = 8.3, 2H), 7.23 (m, 8H), 7.55 (s, 2H), 7.78 (d, *J* = 8.2, 2H), 7.87 (s, 2H). ^13^C-NMR (75 MHz, CDCl_3_): 20.8, 21.1, 22.6, 25.8, 28.1, 40.5, 64.8, 108.8, 123.1, 123.3, 126.5, 127.0, 127.7, 129.9, 133.5, 135.4, 137.1, 140.2, 150.0. 150.7. HRMS *m*/*z* (EI-MS): 643.3418; calcd. for C_44_H_43_N_4_O [M + H]^+^, 643.3431.

*4,7-Bis(9-(p-tolyl)-2,3,4,4a,9,9a-hexahydro-1H-carbazol-6-yl)benzo[c][1,2,5]thiadiazole* (**1b**)

Orange solid, yield 162 mg (82%), m.p. 183–185 °C. R_f_ = 0.53 (hexane:ethylacetate, 10:1, (*v*/*v*)). IR, v_max_, cm^−1^: 2925, 2853, 1606, 1512, 1469, 1343, 1269, 808. ^1^H-NMR (300 MHz, CDCl_3_): 1.48–1.41 (m, 4H), 1.62–1.53 (m, 4H), 1.98–1.70 (m, 8H), 2.39 (s, 6H), 3.37 (m, 2H), 4.18 (m, 2H), 6.89 (d, *J* = 8.2, 2H), 7.23 (s, 8H), 7.70 (s, 1H), 7.72 (d, *J* = 7.4, 2H), 7.78 (s, 2H). ^13^C-NMR (75 MHz, CDCl_3_): 20.9, 21.2, 22.7, 25.9, 28.2, 40.6, 64.7, 108.8, 122.9, 124.1, 126.8, 128.1, 128.4, 129.1, 132.5, 133.2, 135.1, 140.5, 149.4, 154.4. HRMS *m*/*z* (EI-MS): 658.3122; calcd. for C_44_H_43_N_4_S [M + H]^+^, 658.3125.

*4,7-Bis(9-(p-tolyl)-2,3,4,4a,9,9a-hexahydro-1H-carbazol-6-yl)benzo[c][1,2,5]selenadiazole* (**1c**)

Dark red solid, yield 169 mg (80%), m.p. 150–152 °C. R_f_ = 0.52 (hexane:ethylacetate, 5:1, (*v*/*v*)). IR, v_max_, cm^−1^: 2926, 2853, 1605, 1512, 1465, 1376, 1265, 808. ^1^H-NMR (300 MHz, CDCl_3_): 1.47–1.39 (m, 2H), 1.63–1.53 (m, 2H), 1.93–1.71 (m, 4H), 2.40 (s, 6H), 3.37 (m, 2H), 4.18 (m, 2H), 6.90 (d, *J* = 8.2, 2H), 7.24 (s, 8H), 7.56 (s, 2H), 7.64 (d, *J* = 8.2, 2H), 7.71(s, 2H). ^13^C-NMR (75 MHz, CDCl_3_): 20.9, 21.2, 22.8, 25.9, 28.2, 40.6, 64.7, 108.6, 122.9, 124.3, 127.3, 128.6, 128.7, 129.8, 133.1, 134.3, 135.0, 140.6, 149.3, 160.2. HRMS *m*/*z* (EI-MS): 706.2565; calcd. for C_44_H_42_N_4_Se [M]^+^, 706.2537.

## 4. Conclusions

Herein, we report novel bright luminescence dyes based on 9-(*p*-tolyl)-2,3,4,4a,9,9a-hexahydro-1*H*-carbazole as a donor and 2,1,3-benzochalcogenadiazoles as acceptor units. These dyes exhibit bright luminescence originating from an intramolecular charge transfer (ICT) from indoline—9-(*p*-tolyl)-2,3,4,4a,9,9a-hexahydro-1*H*-carbazole donor moiety to 2,1,3-benzochalcogenadiazole acceptor. The influence of the chalcogen atom (oxygen, sulfur, and selenium) in the acceptor unit on the fluorescence properties was estimated. It was shown that the replacement of the light oxygen heteroatom by heavier sulfur in the acceptor moiety leads to a small redshift of the emission maximum, without a notable decrease in luminescence efficiency (about 2–5%) for solutions **1a** and **1b** in various solvents. Excellent electroluminescent performance of dyes **1**(**a**–**c**) in solution-processed multilayer organic light-emitting diodes (OLEDs) was obtained.

## Figures and Tables

**Table 1:** Suzuki–Miyaura coupling of 4,7-dibromo-2,1,3-benzochalcogenadiazoles **2**(**a**–**c**) with 4,6-(4,4,5,5-tetramethyl-1,3,2-dioxaborolan-2-yl)-9-(*p*-tolyl)-1,2,3,4,4a,9a-hexahydrocarbazole **3**.

Entry	Solvent	Dibromide	Temp. (°C)	Time (h)	Yields (%)
1	THF	**2a**	66	10	**1a** (25)
2	THF	**2b**	66	10	**1b** (20)
3	THF	**2c**	66	10	**1c** (22)
4	Dioxane	**2a**	101	24	**1a** (85)
5	Dioxane	**2b**	101	24	**1b** (82)
6	Dioxane	**2c**	101	24	**1c** (80)

**Table 2:** Electrochemical properties of the dyes **1**(**a**–**c**) in DMF solution.

Compound	E^red^ (vs Fc/Fc^+^) ^1^, V	E_LUMO_ ^2^, eV	E^ox^ (vs Fc/Fc^+^) ^1^, V	E_HOMO_ ^2^, eV	Eg ^3^, eV
**1a**	−1.80	−3.30	0.25	−5.35	2.05
**1b**	−1.88	−3.22	0.26	−5.36	2.14
**1c**	−1.59	−3.51	0.22	−5.32	1.81

^1^ Here, E^ox^ and E^red^ are oxidation and reduction peak potential relative to Fc/Fc^+^, respectively. ^2^ Energies of frontier orbitals were calculated according to Equations (1) and (2). ^3^ E_g_ = E_LUMO_ − E_HOMO_.

**Table 3:** Photophysical parameters obtained for **1b**: absorption maximum wavelength, λ_abs_; maximum molar extinction coefficient, ε_max_; wavelength of emission maximum, λ_em_; full width half maximum, FWHM; Stokes shift, Δν; quantum yield, Φ; radiative and non-radiative rate constant, K_r_ and K_nr_, respectively; fluorescence lifetime, τ; oscillator strength, f; transition dipole moment, µ_eg_.

Solvent	λ_abs_ nm	ε_max_ × 10^4^ mol 1^−1^ cm^−1^	λ_em_ nm	FWHM nm	Δν Cm^−1^	Φ %	K_r_ × 10^7^ S^−1^	K_nr_ × 10^8^ S^−1^	τ ns	f	μ_eg_ Debye
cyclohexane	485	10	594	90	3784	56	-	-	-	0.68	2.1
CHCl_3_	437	22	610	121	6490	34	9.1	1.8	3.7	0.82	8.1
AcOEt	470	6	660	132	6125	23	3.3	1.1	6.9	0,78	8.0
THF	470	11	662	126	6170	21	3.1	1.2	6.8	0.72	7.8
DMSO	484	12	761	160	7520	11	5.5	4.5	2.0	0.50	6.0

**Table 4:** Comparative experimental and computational photophysical parameters. Calculated energies of LUMO (E_LUMO_) and HOMO (E_HOMO_): ^a^ computational absorption maximum wavelength, ^b^ experimental absorption maximum wavelength, ^c^ experimentally obtained first excited singlet state S_1_ energy, ^d^ computational oscillator strength of vertical absorption and experimental oscillator strength values, ^e^ computational and experimental transition dipole moment values, ^f^ computational emission maximum wavelength, and ^g^ experimental emission maximum wavelength.

Comp.	E_LUMO_ eV	E_HOMO_ eV	λ_abs_ ^a^ nm	λ_abs_ ^b^ nm	S_1_ ^c^ nm	$f_{calc}/{\;f}_{exp}^{\;d}\;$	$\mu_{calc}/{\;\mu }_{exp}^{\;e}\;\;D$	Major Contribution	λ_em_ ^f^ nm	λ_em_ ^g^ nm
**1a**	−0.34	−6.18	578.5	480	543	1.07/0.92	3.02/9.67	H→ L 93% H-2→ L 5%	551.8	660
**1b**	−0.95	−6.15	564.3	470	559	0.83/0.71	4.88/7.80	H→ L 93% H-2→ L 5%	715	662
**1c**	−1.02	−6.14	590.8	495	567	0.68/0.83	5.41/9.33	H→ L 93% H-2→ L 6%	765	705

## Supplementary Materials

The following are available online, Figure S1: Cyclic voltammograms of compounds; Figure S2: UV–Vis spectra for solutions of **1b**; Figure S3: Experimental determination of S1 energies for **1**(**a**–**c**); Figure S4: PL decays for **1b**.

## Sample Availability

Samples of the compounds **1**(**a**–**c**) are available from the authors.
