# Supplementary material for: Novel D-A-D Fluorescent Dyes Based on 9-(p-Tolyl)-2,3,4,4a,9,9a-hexahydro-1H-carbazole as a Donor Unit for Solution-Processed Organic Light-Emitting-Diodes
Source: Molecules. 2021 May 12;26(10):2872. doi: 10.3390/molecules26102872 (PMC8151704; doi:10.3390/molecules26102872)
Supplement: Supplementary file 1 [file molecules-26-02872-s001.zip › molecules-1184436-supplementary.pdf]

Supporting Information

A novel D-A-D fluorescent dyes based on 9-(*p*-tolyl)-2,3,4,4a,9,9a-hexahydro-1*H*-carbazole as a donor unit for solution-processed organic light-emitting-diodes

Vladislav M. Korshunov[a,b*], Maxim S. Mikhailov[c,d], Timofey N. Chmovzh[c,d], Andrey A. Vashchenko[a], Nikita S. Gudim[c], Lyudmila V. Mikhalchenko[c], Ilya V. Taydakov[a], and Oleg A. Rakitin[c,d*]

[a]*P. N. Lebedev Physical Institute of the Russian Academy of Sciences, 53 Leninskiy Prospect, 119991 Moscow, Russia*

[b]*Bauman Moscow State Technical University, 2-ya Baumanskaya Str. 5/1, 105005 Moscow, Russia*

[c]*N. D. Zelinsky Institute of Organic Chemistry, Russian Academy of Sciences, Leninsky Prospect, 119991 Moscow, Russia*

[d]*Nanotechnology Education and Research Center, South Ural State University, 454080 Chelyabinsk, Russia*

E-mails: vladkorshunov@bk.ru, orakitin@ioc.ac.ru

Table of contents:

^{1}H and ^{13}C NMR spectra……………………………………………………......S2
Cyclic voltammograms………………………………………………………..........S5
Photophysical parameters…………………………………………………………S5

^{1}H and ^{13}C NMR spectra

4,7-Bis(9-(p-tolyl)-2,3,4,4a,9,9a-hexahydro-1H-carbazol-6-yl)benzo[c][1,2,5]oxadiazole (1a)

^{1}H NMR (300 MHz)

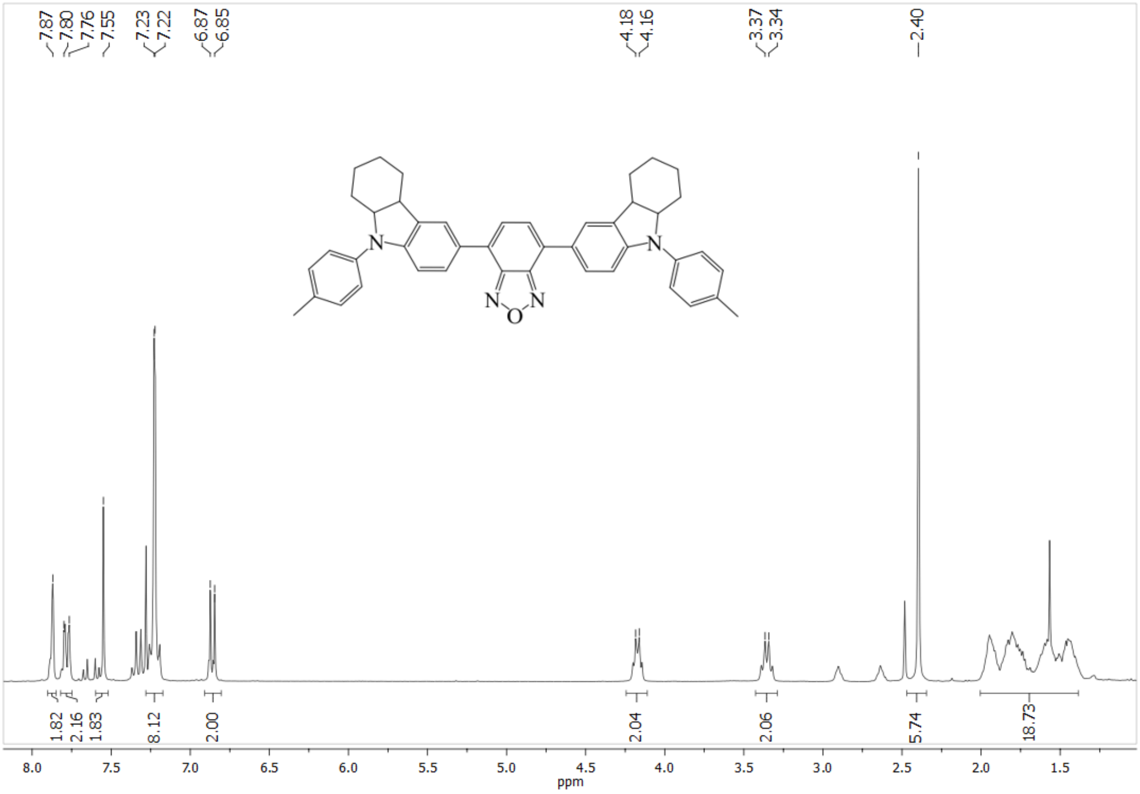

^{13}C NMR(75 MHz)

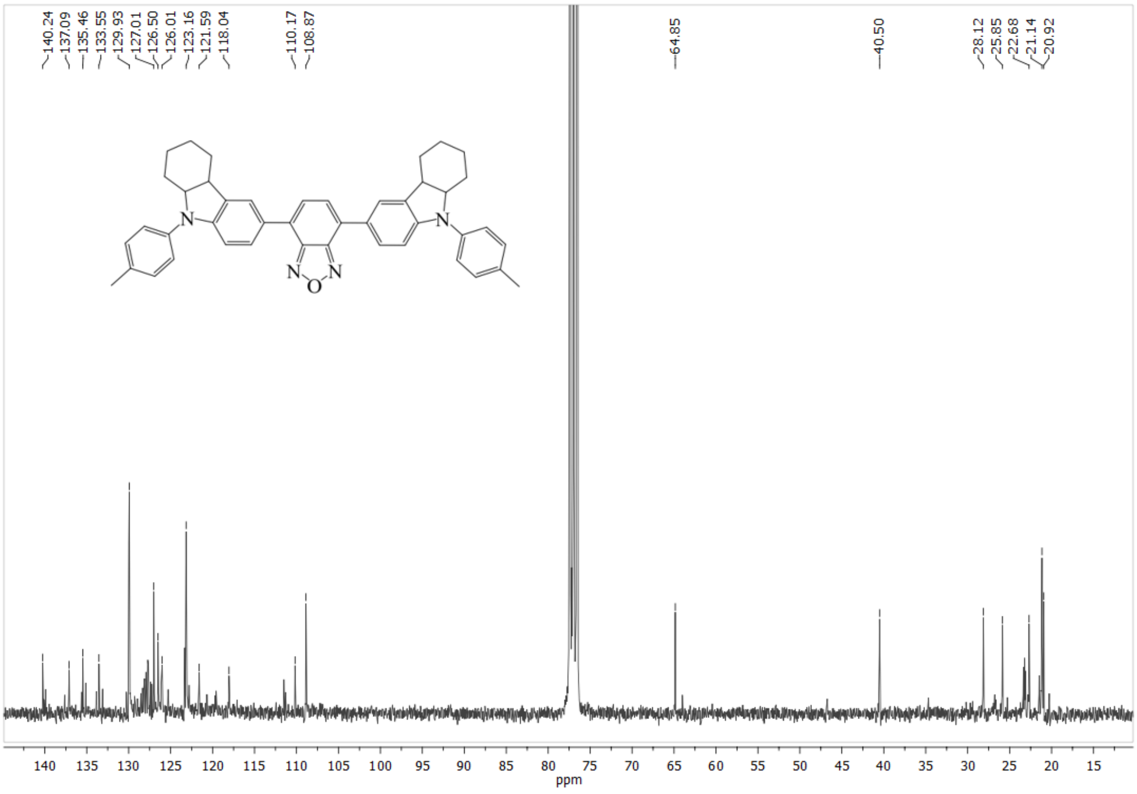

4,7-Bis(9-(p-tolyl)-2,3,4,4a,9,9a-hexahydro-1H-carbazol-6-yl)benzo[c][1,2,5]thiadiazole (1b)

^{1}H NMR (300 MHz)

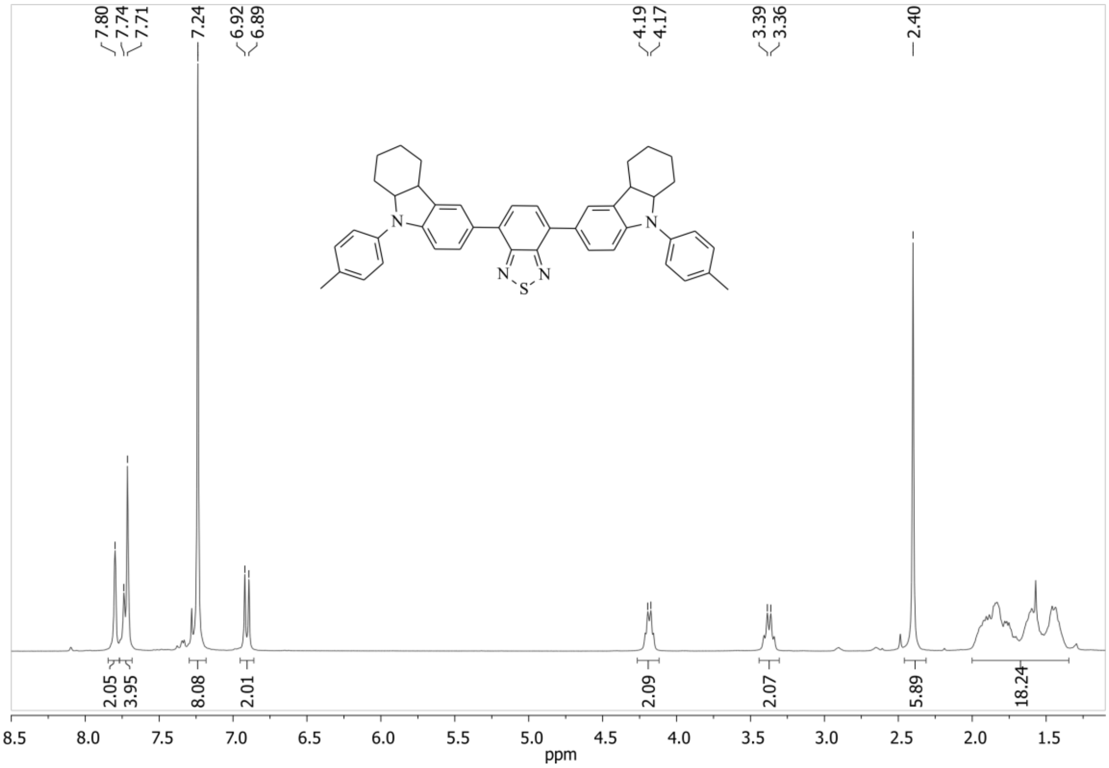

^{13}C NMR(75 MHz)

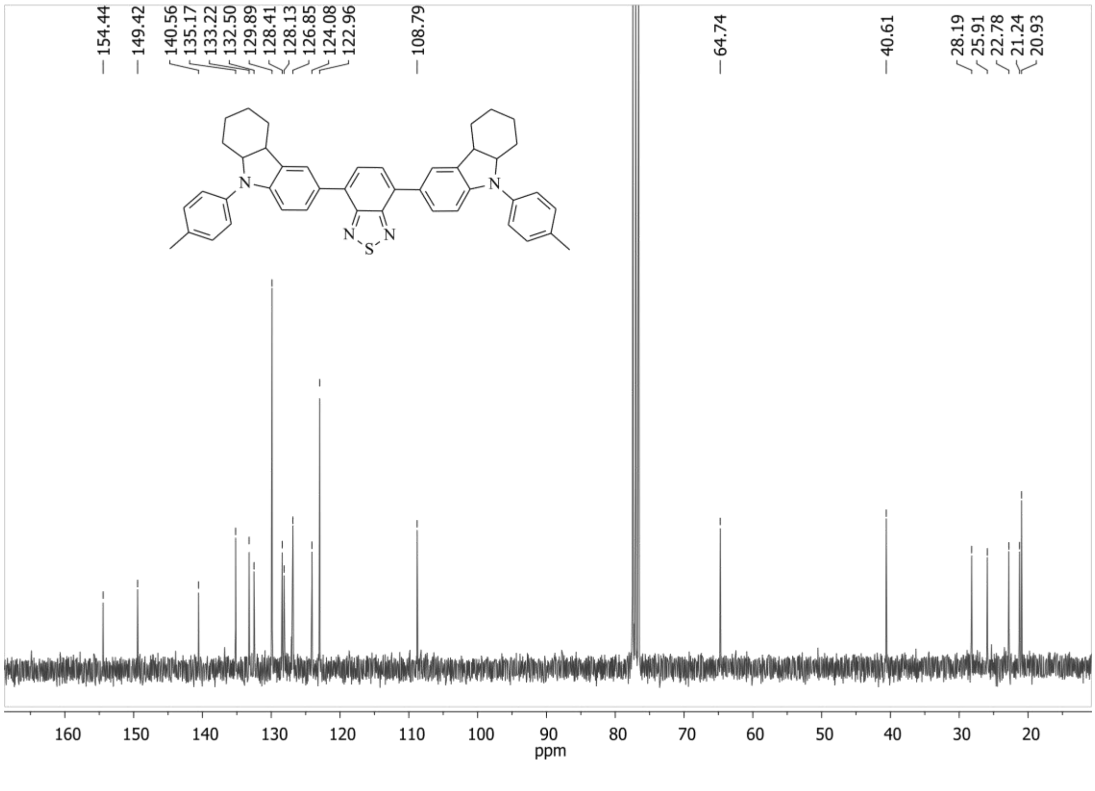

4,7-Bis(9-(p-tolyl)-2,3,4,4a,9,9a-hexahydro-1H-carbazol-6-yl)benzo[c][1,2,5]selenadiazole (1c):

^{1}H NMR (300 MHz)

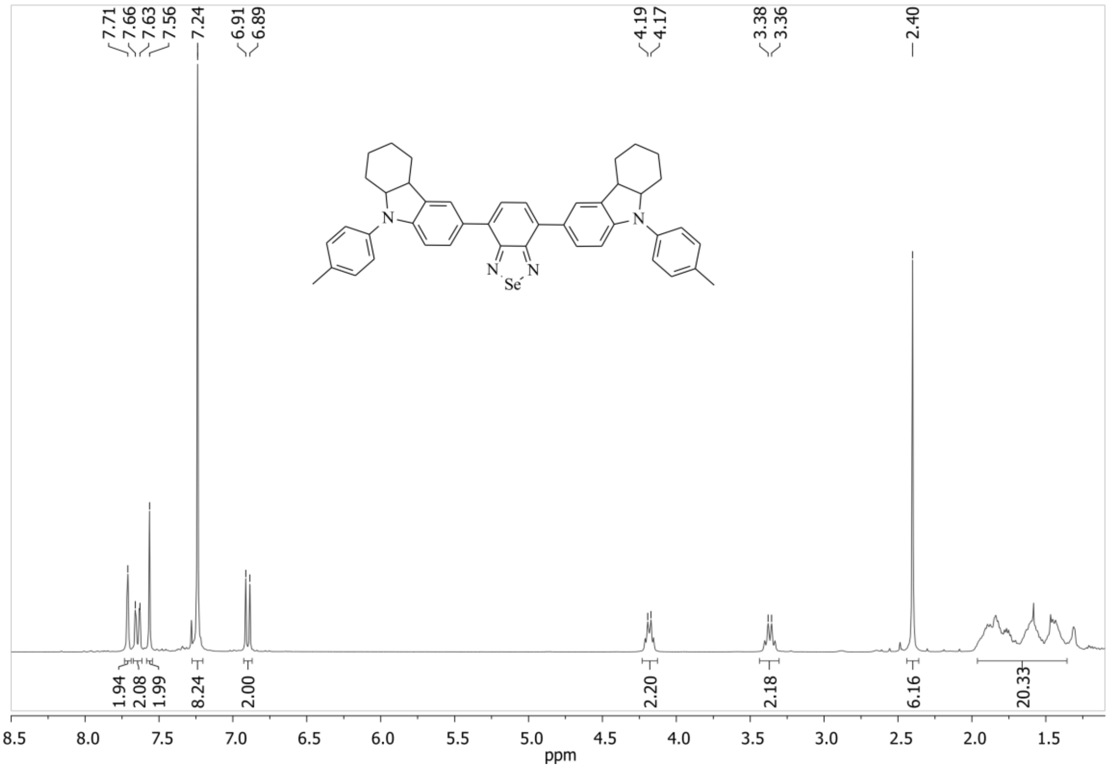

^{13}C NMR(75 MHz)

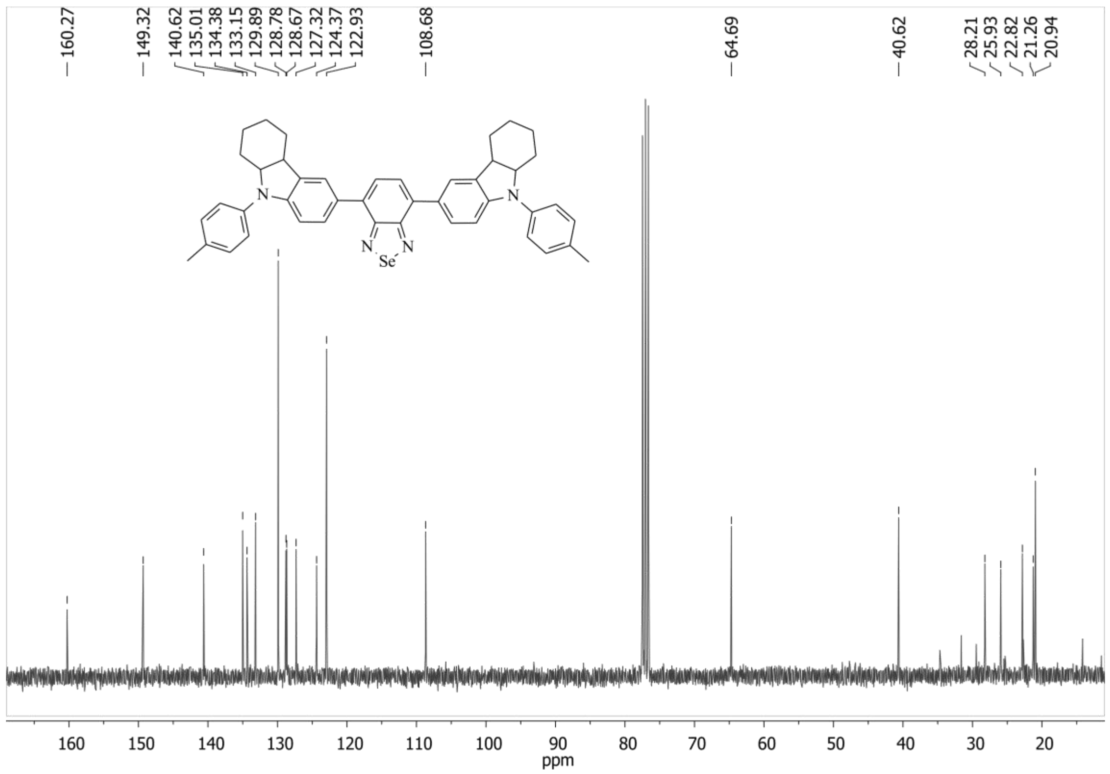

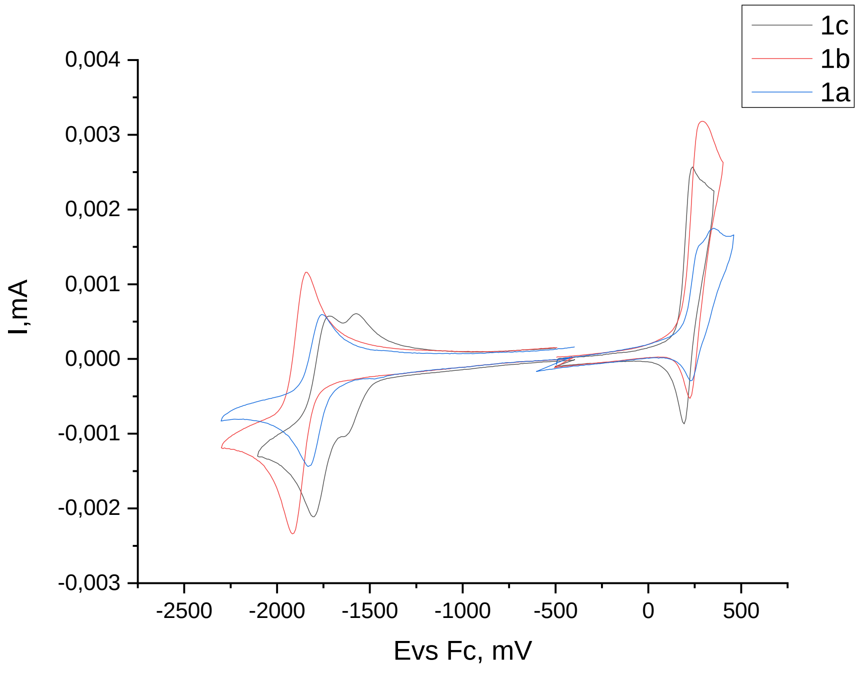

Figure S1: Curves of electroreduction and electrooxidation of compounds **1(a-c)** on a platinum electrode at a potential deposition rate of 0.1 Vs^{-1} in DMF containing 0.1 M Bu_4NBF_4 as a supporting electrolyte.

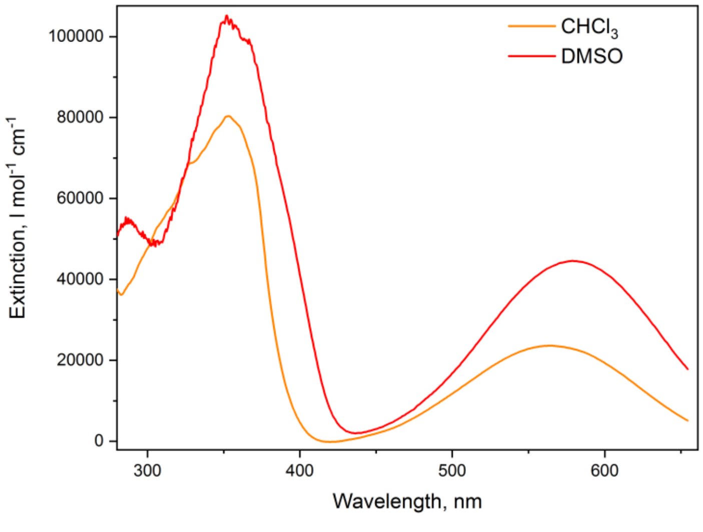

Figure S2: UV-Vis spectra for solutions of **1b**.

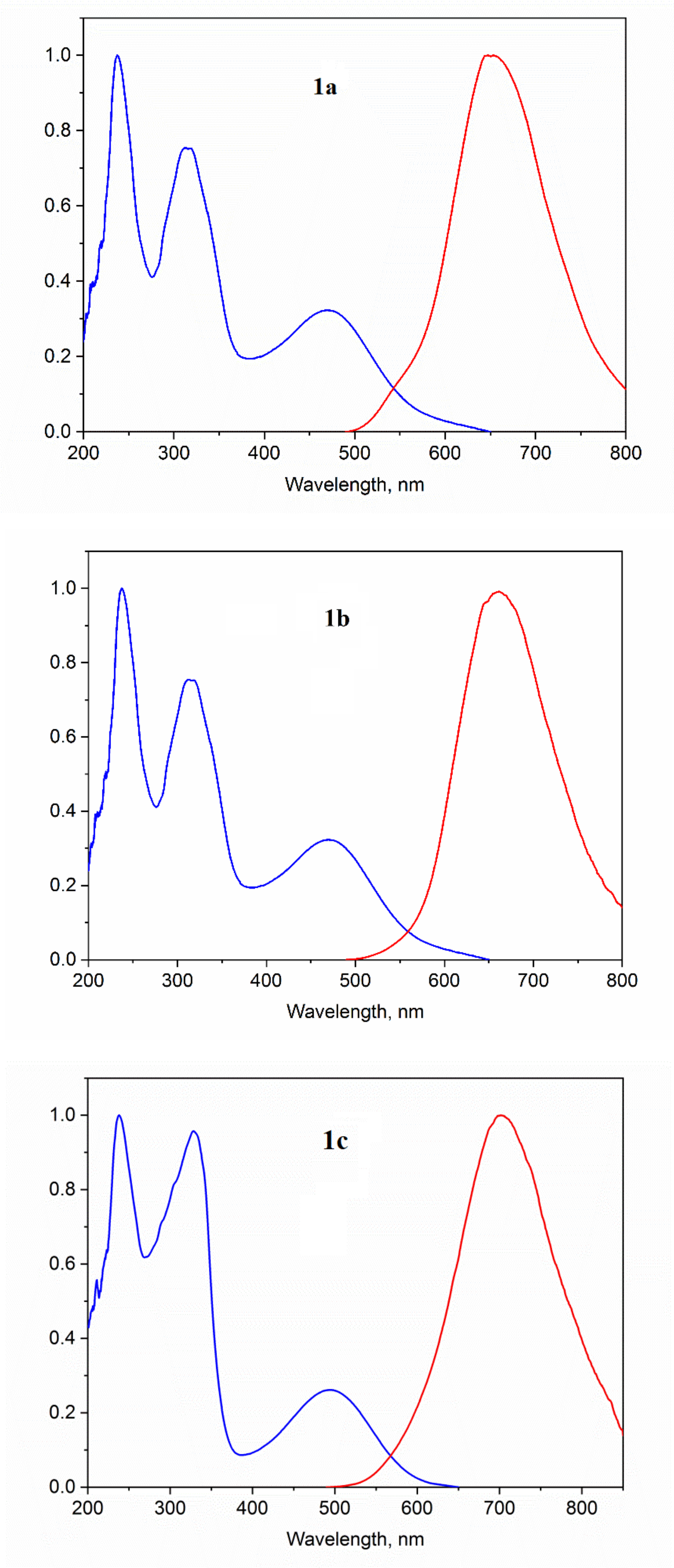

Figure S3: Experimental determination of S_1 energies for **1**(**a-c**): absorption spectra and PL spectra normalized to 1.

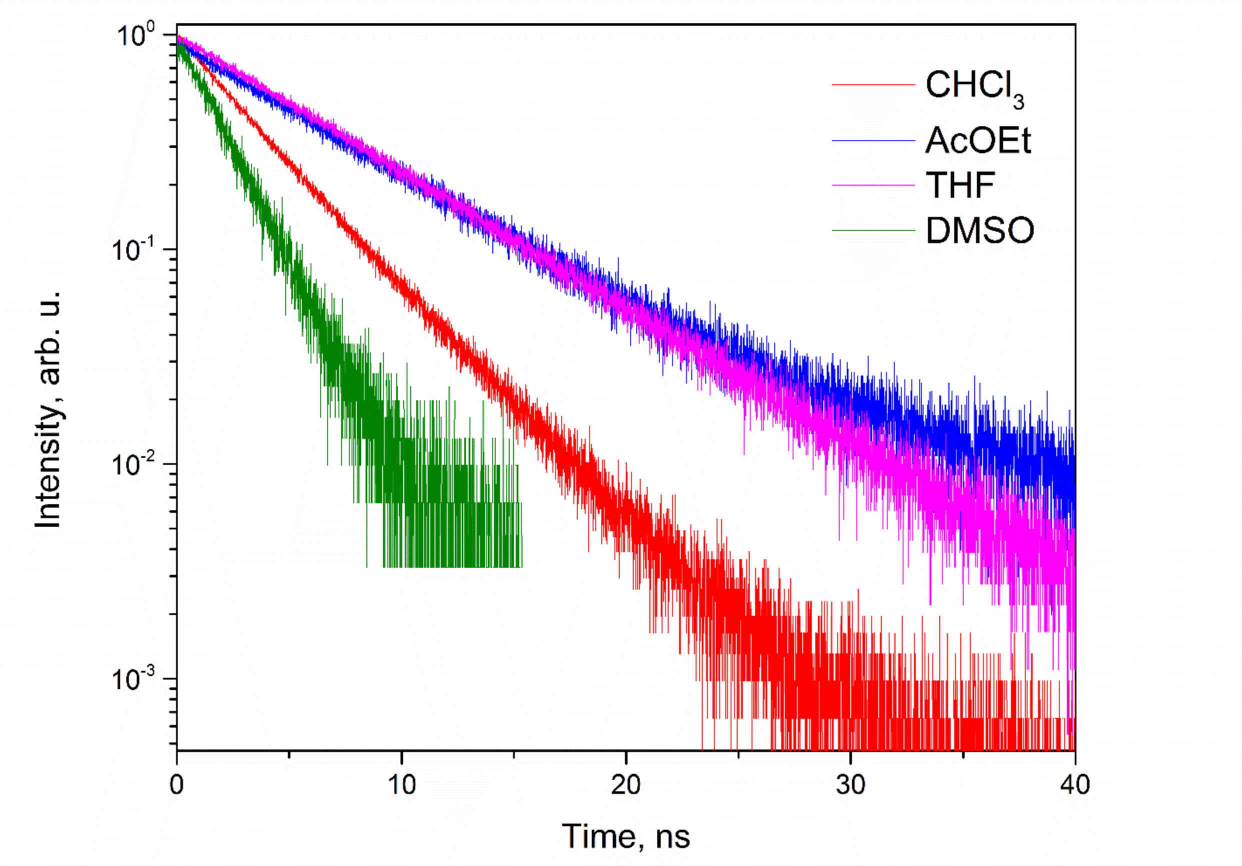

Figure S4: PL decays for **1b**.
